# Foveal cone loss in tamoxifen maculopathy: a case report

**DOI:** 10.1186/s13256-023-04199-z

**Published:** 2023-11-08

**Authors:** Nathan Doble, Elaine M. Wells-Gray, Michael Wells, Stacey S. Choi

**Affiliations:** 1 College of Optometry, The Ohio State University, 338 W 10Th Ave, Columbus, OH 43210 USA; 2 Department of Ophthalmology and Vision Science, Havener Eye Institute, The Ohio State University, 915 Olentangy River Road, Columbus, OH 43212 USA; 3 Present Address: Lumata Health, 1111 N. Lee Ave., Suite 210, Oklahoma, OK 97103 USA

**Keywords:** Adaptive optics, Cone photoreceptors, Imaging, Retina, Scanning laser ophthalmoscopy, Tamoxifen maculopathy

## Abstract

**Background:**

Tamoxifen is used in low dose concentrations (20–40 mg per day) as a therapy for breast cancer but is known to have ocular side effects. In this case report, the foveal cone integrity in a tamoxifen-treated patient who complained of a small central scotoma in the left eye while reading was examined using high resolution adaptive optics imaging.

**Case presentation:**

Both eyes of a 54-year-old Caucasian, non-hispanic female who had been treated with tamoxifen for 1.5 years were examined using various imaging modalities including fundus photography, fundus autofluorescence, fluorescein angiography, spectral-domain optical coherence tomography, and adaptive optics scanning laser ophthalmoscopy. Clinical spectral-domain optical coherence tomography showed a very small disruption to the photoreceptor layer at the fovea in the left eye only. However, adaptive optics scanning laser ophthalmoscopy imaging revealed foveal cone loss in both eyes, but to a lesser extent in the right eye. Inner retinal changes were not observed in either eye.

**Conclusion:**

The area of cone loss was similar in size to a single newsprint letter when projected onto the retina, matching the patient’s description of a scotoma in the left eye. Given the isolated loss of foveal cone photoreceptors with the absence of previously reported inner retinal and vascular changes, our results may indicate the earliest retinal changes associated with tamoxifen retinopathy.

## Background

Tamoxifen is used in low dose concentrations (20–40 mg per day) as a therapy for breast cancer patients. Clinical ocular imaging has revealed side effects including crystalline deposits in the nerve fiber and inner plexiform layers [1–5], pseudocystoid changes at the fovea [1, 2, 6–8], as well as loss of blood flow in the deep capillary plexus [9]. This report describes the high-resolution imaging findings on a tamoxifen patient who complained of a small central scotoma in the left eye while reading. However, subtle loss of cone photoreceptors was also observed in the right eye, which was not visible in standard clinical imaging.

## Case presentation

A 54-year-old Caucasian, non-hispanic female complained of a small central scotoma in the left eye equal to the size of a printed character while reading. The patient had been previously diagnosed with lobular carcinoma *in situ* (LCIS) for which she was prescribed tamoxifen (20 mg daily), and she continued for a period of 1.5 years (cumulative dose of 10.95 g); after that period, the treatment was discontinued. The patient subsequently underwent a lumpectomy 5 months later but did not have chemotherapy or radiation therapy. No baseline ocular examination results were available before the start of the tamoxifen treatment. It was noted that the patient was not on any other medications during or after the tamoxifen treatment.

The patient had undergone laser-assisted *in situ* keratomileusis (LASIK) treatment previously, but had no other confounding ocular conditions. Her current prescription was −1.25 + 0.25 × 2 (OD) and +2.75 + 0.25 × 131 (OS), and the best corrected visual acuity was 20/20 in each eye. Humphrey visual fields (30-2 and 10-2 SITA-standard threshold tests) were performed. Both tests were normal with good test reliability and hence were not repeated.

**Fig. 1:**
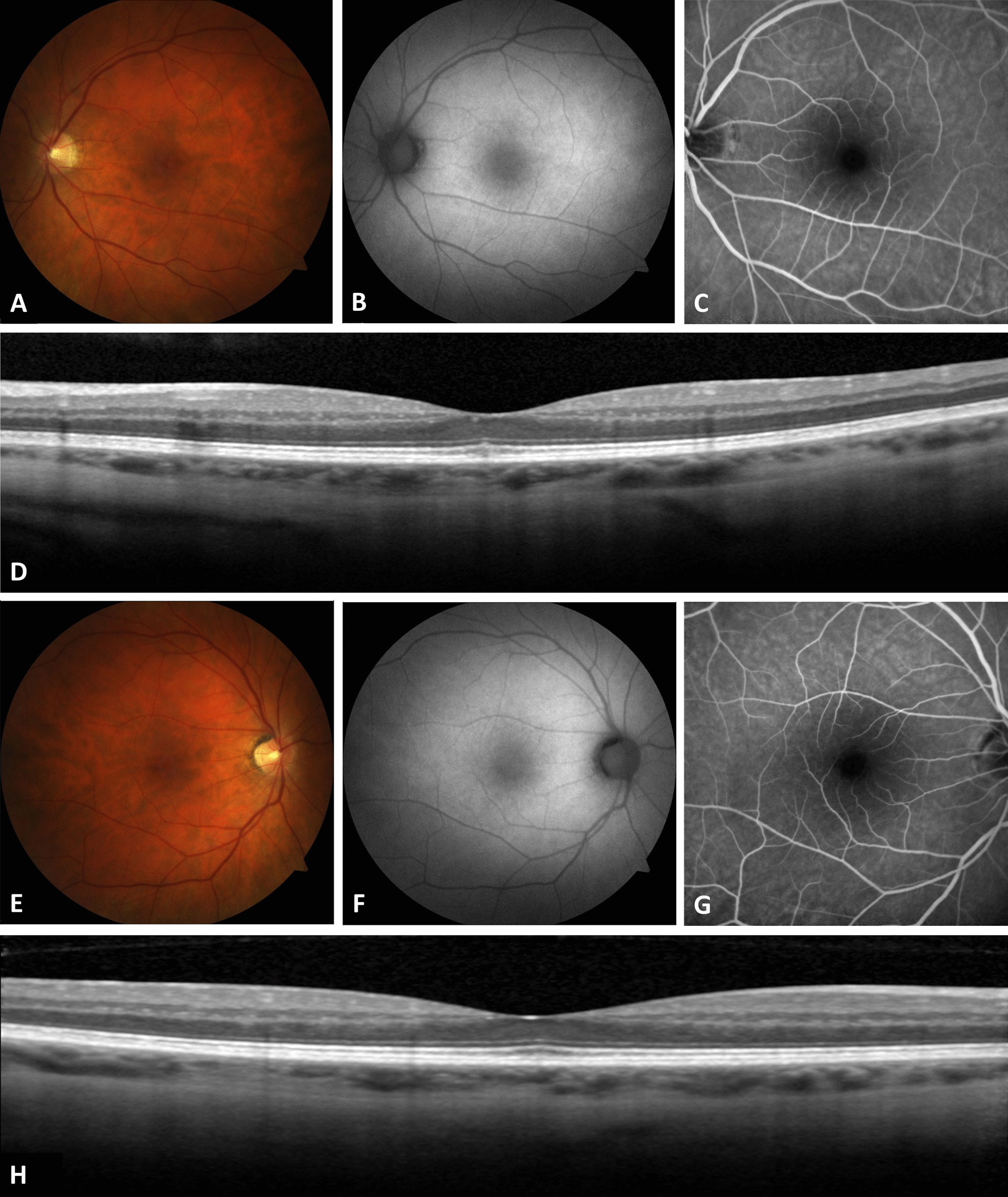
**A** Fundus photography (FP), **B** fundus autofluorescence (FAF), and **C** fluorescein angiography (FA) for the left eye were unremarkable. **D** Spectral domain-optical coherence tomography (SD-OCT) of the left eye showing intact outer retinal layers outside of the fovea. However, at the fovea, there is a disruption to the photoreceptor layer with a hyper-reflective area in the cone inner segment (IS) layer. The external limiting membrane (ELM) is intact. The FP, FAF, and FA, for the right eye, **E**–**G**, respectively, were also unremarkable. **H** SD-OCT of the right eye showing normal macular structure; both the inner and outer retinal layers are contiguous and normal throughout the B-scan. Both SD-OCT B-scans are 6 mm in length

A dilated fundus examination revealed a duller foveal light reflex and a yellowish foveal discoloration in the left eye. However, fundus photography (FP), fundus autofluorescence (FAF), and fluorescein angiography (FA) (Fig. 1A–C) were unremarkable. FP, FAF, and FA for the right eye were also unremarkable (Fig. 1E–G). The FAF images for either eye did not show any areas of abnormal hyper- or hypo-autofluorescence. All other findings including optic discs, vessels, and peripheral retina were unremarkable in both eyes.

Figure 1D shows the spectral spectral-domain optical coherence tomography (SD-OCT) image from the left eye showing focal disruption of cone outer segment (OS) layer at the foveal center with a localized hyper-reflective region located in the cone inner segment (IS) layer. The external limiting membrane (ELM) appears intact. The SD-OCT for the right eye shows a normal macula with contiguous outer retinal layers (Fig. 1H). The inner retinal layers appear to be normal across the retina for both eyes. Both FAF and SD-OCT images were acquired using Spectralis OCT (Heidelberg Engineering Inc., Heidelberg, Germany).

After observing the subtle foveal irregularities in the photoreceptor layer in the left eye from the SD-OCT image, the patient was imaged on a research-grade AO-SLO system. The system hardware and imaging procedure are described in detail in Wells-Gray *et al.* [10]. Briefly, the AO-SLO acquires 1° × 1° images of the retina at 60 frames per second at an imaging wavelength of 680 nm with a lateral resolution of ~ 2.3 µm. Prior to imaging, a combination of 1% tropicamide and 2.5% phenylephrine was used to dilate the pupil and paralyze accommodation. During imaging, the patient looked at a fixation target displayed on a computer monitor, which corresponded to the area of imaging. Multiple AO-SLO datasets were acquired around the foveal center of both eyes and subsequently postprocessed to remove eye motion. For the AO imaging, the tenets of the Declaration of Helsinki were observed, and the protocol was approved by the Institutional Review Board of The Ohio State University (OSU). Written informed consent was obtained after all procedures were fully explained to the patient and prior to imaging.

**Fig. 2:**
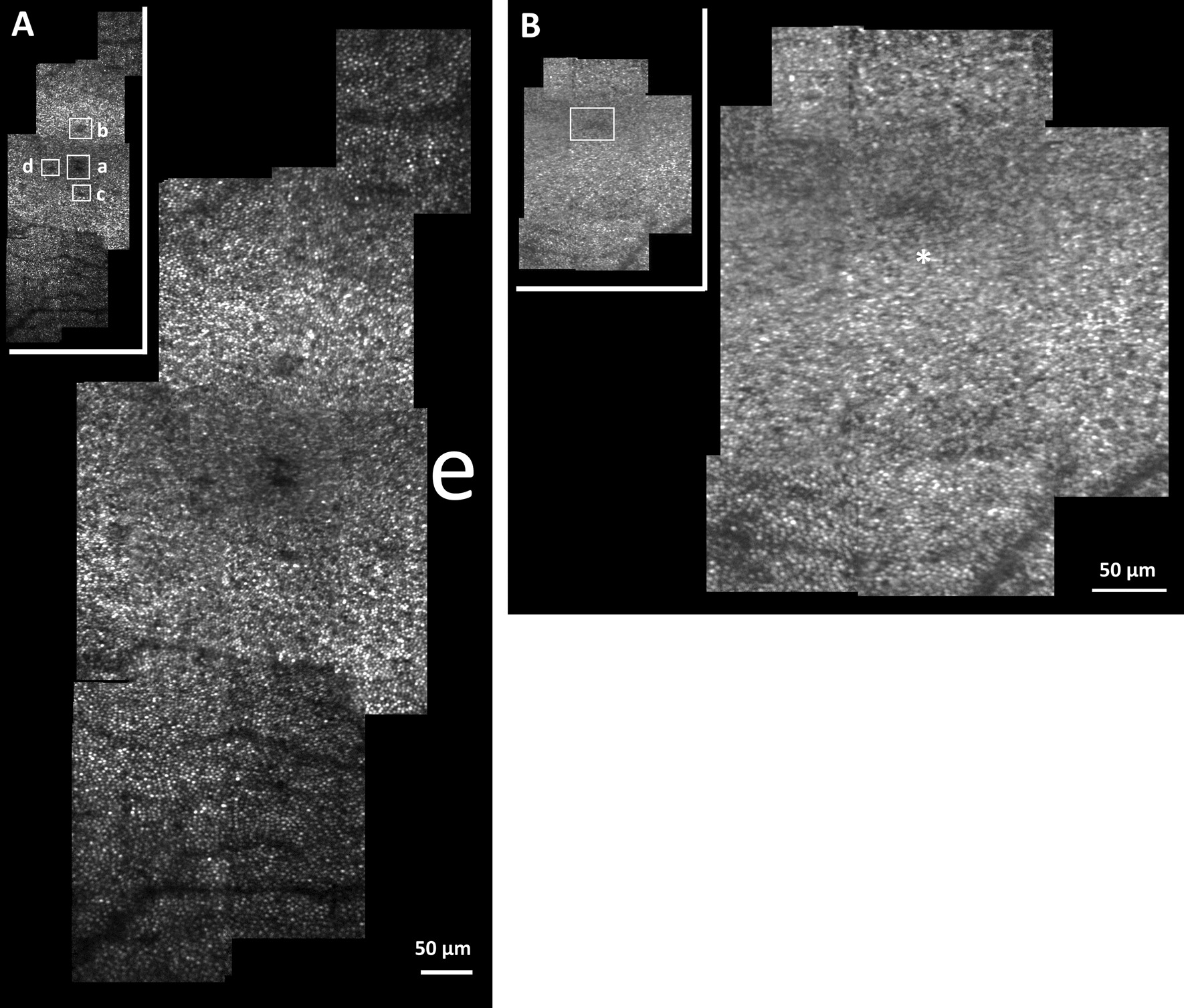
**A** Montage of the adaptive optics-scanning laser ophthalmoscope (AO-SLO) images from the affected left eye, field of view is 1.4° × 2.8° (380 × 760 µm) centered on the fovea. The inset figure in the top left shows the location of the lesions (**a**–**d**) highlighted by the white bounding boxes. The lesion (**a**) is comprised of two smaller lesions and are located at the foveal center. Two additional smaller lesions (**b**, **c**) are located 75–100 µm from the foveal center in the superior and inferior retina, respectively. Several smaller and less distinct lesions (**d**) were also observed ~75 µm horizontally from (**a**). Shown for comparison is a scaled “e” representative of letter size while reading newsprint at a distance of 40 cm. **B** Montage of the AO-SLO images from asymptomatic right eye, 1.1° × 1.2° (298 × 314 µm). The inset figure in the top left shows the location of the lesion highlighted by the white bounding box. While not as visible as in the left eye, a small central, foveal lesion appears to be present just superior to the asterisk

The montage of AO-SLO images for the left and right eyes, respectively, are shown (Fig.2A, B). Lesions are more clearly visible in the left eye as compared with the right. Cone densities (using a 50 × 50 µm window) measured at (i) fovea, (ii) 75 µm superior, and (iii) 150 µm superior from the foveal center (just outside of the lesion region) were 26,000, 29,600 and 53,200 cones/mm^2^, respectively, for the affected left eye. The measured values close to the foveal center are much lower than the > 100,000 cones/mm^2^ expected from the literature [11], however the result at 150 µm from the foveal center is in good agreement with the expected value for age-matched healthy subjects [12]. For the right eye, the foveal cone density was 39,200 cones/mm^2^, greater than that measured in the left eye but still well below the expected value for an age-matched control. Cone densities at 75 µm superior and 150 µm superior from the foveal center were 52,000 and 58,400 cones/mm^2^, respectively, in good agreement with published results [11].

## Discussion

Previous SD-OCT studies have shown disruption to the photoreceptor layer in association with tamoxifen use, which is in agreement with the findings presented here here [1, 2, 6–8, 13, 14]. While inner retina deposits and vascular changes have also been reported [1–5, 9], these were not observed in our case. Only the loss of foveal cones was revealed in both the symptomatic (left) and asymptomatic (right) eyes using the AO-SLO, and only in the symptomatic eye using a clinical OCT system.

In our patient, all the layers anterior and posterior to the photoreceptor layer appeared to be regular and intact based on the SD-OCT images. Perifoveal telangiectasia was discounted based on the normal fluorescein angiography. While the high-resolution AO-SLO imaging of the lesions did show areas of foveal cone loss, the cone photoreceptor mosaic 150 µm from the foveal center for the left eye and outside the umbo (right eye) were normal, as shown by the cone density measurements.

The subject had previously undergone LASIK surgery, but, unfortunately, their refractive error prior to this surgery was not known. It is well established that myopic eyes have reduced cone density compared with their age-matched emmetropic or hyperopic eyes primarily due to the retinal stretching caused by the axial elongation [15]. Any pre-LASIK myopia would not explain the findings reported here as the cone density changes were limited to the center of the fovea only and normal cone densities were found immediately outside this area.

Solar retinopathy [16] was discounted, as one would expect the retinal changes to be more or less similar in both eyes and for the typical hyper-reflectivity observed in such cases to span multiple retinal layers. Furthermore, FAF and FA for both eyes were unremarkable contrary to what is typically found in solar retinopathy. [17]

To explain the patient’s complaint when reading, lesion size was compared to standard letter size while reading newsprint (Fig. 2A). When compared to a scaled letter “e” representative of text size [18] while reading at a distance of 40 cm, it appears the central lesion in the left eye is large enough to obscure single letters while reading, potentially explaining her central scotoma.

## Conclusion

The pathophysiology of tamoxifen maculopathy is yet to be understood, but it is thought to be related to dysfunction and degeneration of Müller cells [19]. Given the isolated loss of foveal cone photoreceptors with the absence of previously reported inner retinal and vascular changes, our results may indicate the earliest changes associated with tamoxifen retinopathy. Furthermore, SD-OCT failed to show any structural changes in the asymptomatic eye (right eye) while the AO-SLO was able to detect smaller focal areas of cone loss, demonstrating the ability to detect structural changes before manifestation of functional loss noticed by the patient.

To our knowledge, this is the first report showing the *in vivo* loss of individual foveal cone photoreceptors at the fovea in tamoxifen maculopathy at a resolution not possible with current clinical instrumentation.

## Data Availability

The datasets used and/or analyzed during the current study are available from the corresponding author on reasonable request.

## Abbreviations

LCIS: Lobular carcinoma *in situ*
LASIK: Laser-assisted *in situ* keratomileusis
FAF: Fundus autofluorescence
FP: Fundus photography
FA: Fluorescein angiography
OCT: Optical coherence tomography
SD-OCT: Spectral-domain optical coherence tomography
OS: Outer segment
IS: Inner segment
ELM: External limiting membrane
AO-SLO: Adaptive optics scanning laser ophthalmoscopy
SITA: Swedish interactive thresholding algorithm
OSU: The Ohio State University
